# Mortality Among Dental Healthcare Workers During the Coronavirus Disease 2019 Pandemic: A Public Domain Database Study

**DOI:** 10.1016/j.identj.2024.10.012

**Published:** 2024-12-18

**Authors:** Marlotte C. van Capelleveen, Thérèse A. Elkerbout, Eveline van der Sluijs, Nadine Jaquet, Dagmar Else Slot

**Affiliations:** Department of Periodontology, Academic Centre for Dentistry Amsterdam (ACTA), A Joint Venture Between the Faculty of Dentistry and University of Amsterdam and the Faculty of Dentistry of the Vrije Universiteit Amsterdam, Amsterdam, The Netherlands

**Keywords:** Dental healthcare workers, Dentist, Dental hygienist, Dental assistant, SARS-CoV-2, COVID-19

## Abstract

**Background:**

This study was conducted to assess the effects of severe acute respiratory syndrome coronavirus 2 (SARS-CoV-2) on dental healthcare workers (DHCWs) during the pandemic. Due to frequent exposure to aerosol-generating procedures, DHCWs are at an increased risk of a SARS-CoV-2 infection, increasing their mortality risk. Therefore, this retrospective study aimed to ascertain the mortality of DHCWs attributable to SARS-CoV-2 infection from databases in the public domain during the pandemic.

**Methods:**

The data were obtained from three public-domain online databases: Medscape, FNOMCeO, and X. All collected data from March 2020 to May 2024 were merged into a tabulated format. An analysis was conducted on the data collectively and through a subgroup analysis based on the country, detailed profession, age, gender, and date of death.

**Results:**

During the SARS-CoV-2 pandemic, 100 DHCWs were deceased. The DHCWs in the study were employed in 14 countries, with the United States (40%) and Italy (34%) listing the highest percentages of deaths of these workers. Dentists constituted 79% of the total DHCWs. The gender distribution among the deceased was 85 (85%) men and 15 (15%) women. The age of 57 deceased DHCWs was known, resulting in a mean age of 58 years (ranging from 28 to 88). The DHCWs at ages 60 to 70 years (49%) exhibited the highest mortality rate from SARS-CoV-2. The date of death was known for 73 DHCWs, who were deceased between March 2020 and February 2023.

**Conclusion and clinical relevance:**

During the global pandemic, DHCWs worked in the context of general practice. The DHCWs adhered to infection prevention protocols according to standard guidelines and incorporated new adjunctive measures, especially for the pandemic, including adopting coronavirus disease 2019 guidelines for oral healthcare. These measures provided satisfactory protection, with less than 0.006% mortality of the estimated DHCWs worldwide.

## Introduction

In November 2019, the novel severe acute respiratory syndrome coronavirus 2 (SARS-CoV-2) was discovered. This virus is responsible for causing the critical disease known as coronavirus disease 2019 (COVID-19).^1^ The first case of COVID-19 emerged in Wuhan, China, and approximately 4 months later, on 11 March 2020, the World Health Organization (WHO) declared COVID-19 a global pandemic.^2^ Over 3 years (from 26 January 2020, to 10 March 2023), COVID-19 resulted in the loss of 6881,955 lives worldwide,^3^ representing approximately 0.09% of the global population.

The coronavirus has resulted in numerous casualties globally due to its high virulence and contagious nature. Person-to-person transmission of viruses, such as SARS-CoV-2, Middle East respiratory syndrome-virus, and influenza, occurs primarily during close contact through respiratory droplets containing viral particles and direct contact with contaminated surfaces.^4^ However, the extensive spread of COVID-19 infections cannot be exclusively attributed to droplet deposition or contact contamination. Evidence suggests that SARS-CoV-2 can also be transmitted through aerosols.^5^ Thus, airborne transmission plays a crucial role in contaminating the host body with SARS-CoV-2.^5^

Individuals infected with SARS-CoV-2 disseminate aerosols (solid and liquid particles), and droplets by coughing, sneezing, and speaking.^6^ This dissemination risks a potential infection in individuals near the infected person, such as healthcare professionals. Evidence demonstrates that nearly one-tenth of healthcare professionals working in hospitals have been diagnosed with SARS-CoV-2.^7^ In addition, WHO estimates that between 80,000 and 180,000 healthcare workers have died from COVID-19.^8^

In oral healthcare, aerosol-generating procedures are frequently performed. Dental aerosols can contaminate the mucous membranes of oral healthcare practitioners (including their oral cavities, respiratory passages, and ocular regions) and the exposed surfaces and materials in the surrounding environment.^9^ Consequently, dental healthcare workers (DHCWs) were at a heightened infection transmission risk during the pandemic. Patients required urgent dental care, such as pain relief, which could only be provided by DHCWs.^10^ According to the *New York Times* on 15 March 2020, the dental hygienist occupation was identified as facing the highest risk of SARS-CoV-2 infection, with dental assistants and dentists closely following.^10^

Thus, it was posited that healthcare workers, particularly DHCWs, are susceptible to contracting COVID-19 through occupational exposure. In 2020, an analysis of reported deaths from COVID-19 among healthcare and social workers was published, albeit limited to the United Kingdom.^11^ Currently, the precise number of DHCWs worldwide who succumbed to the SARS-CoV-2 infection remains unknown. Hence, the extent of the problem remains unclear and warrants further analysis. Therefore, a retrospective evaluation is necessary to ascertain the number of DHCWs who did not survive the pandemic.

## Methods

### Protocol, reporting, and ethics

This study is a retrospective analysis adhering to the RECORD guidelines.^12^ The study protocol was developed a priori, and the Institutional Review Board committee of ACTA approved it with registration number 2022-*52198*. The study complies with the General Data Protection Regulation, as all data were sourced from online public databases.^13^

### Research question

How many DHCWs worldwide died due to the SARS-CoV-2 pandemic as reported in public-domain databases up to May 2024?

### Databases

The data employed in this study were collected from three publicly accessible online platforms: Medscape, FNOMCeO (Portale della Federazione Nazionale degli Ordini dei Medici Chirurghi e degli Odontoiatri), and X (@CTZebra). All three sources aim to pay tribute to the healthcare workers who died because of the COVID-19 pandemic.

The first database, Medscape, comprises a nonscientific layperson press article titled ‘In Memoriam: Healthcare Workers Who Have Died of COVID-19’,^14^ as last updated on 3 June 2021. This article is a tribute to healthcare workers who provided care to COVID-19 patients while exposing themselves to the infection risk. The compilation of healthcare workers whose deaths were related to COVID-19 was facilitated by colleagues, friends, and family members of the deceased. They used an online form to provide details, such as name, age, profession or specialty, location, name of the facility or hospital, and a link to the information source. The provided link was verified to validate the accuracy of the provided data before being published on the website.

The second database comprises a continuously updated Italian nonscientific layperson press article titled ‘Elenco dei Medici caduti nel corso dell'epidemia di COVID-19’ (‘List of doctors who died during the COVID-19 epidemic’), available on the website of FNOMCeO.^15^ The text was translated from Italian to English using Microsoft Edge automatic translation. The article consists of a list of names of Italian doctors, including medical specialists and dentists, along with the date of death due to COVID-19. The list encompasses all doctors, regardless of retirement status, as being a doctor is considered a lifelong commitment, and the pain and loss are perceived equally. Moreover, some doctors who had retired or were on a leave returned to assist in the medical field.

The third database is an X account featuring a memorandum to US healthcare workers who died of COVID-19,^16^ with the latest COVID-19-associated postdating to 23 May 2023. These posts include personal details regarding the deceased individuals, such as photographs, profession, family situation, hobbies, and characteristics.

### Study procedures

This study includes a purposive sample of DHCWs, explicitly targeting the identification of DHCWs deceased due to COVID-19. A focused search for eligible DHCWs was conducted across three sources to compile a comprehensive list of all deceased individuals under this category. The extracted data from the databases encompassed personal details, including name, gender, age, country, specific profession, date of death, and the respective database source. Subject names identified in multiple databases were considered a single entry to avoid duplication. When gender data were unavailable, the artificial intelligence (AI) program Gender-API.com^17^ was used to analyse the names and determine the associated genders.

### Data analysis

The data were analysed using IBM SPSS software for Mac (v28.0, IBM Corporation) between September 2023 and November 2023. Descriptive statistics were applied to summarize the data, focusing on gender, age, country, specific profession, date of death, and data source. The analysis included the evaluation of the month and number of deaths and the combination of age and death. Only known data that were considered in the analysis and instances where age was not reported were excluded from further analysis. The first pandemic wave in March and April of 2020, the second wave in December and January of 2021,^18^ and the moment when vaccines became available starting in January 2021^3^ were analysed regarding the month of death.

## Results

**Fig. 1:**
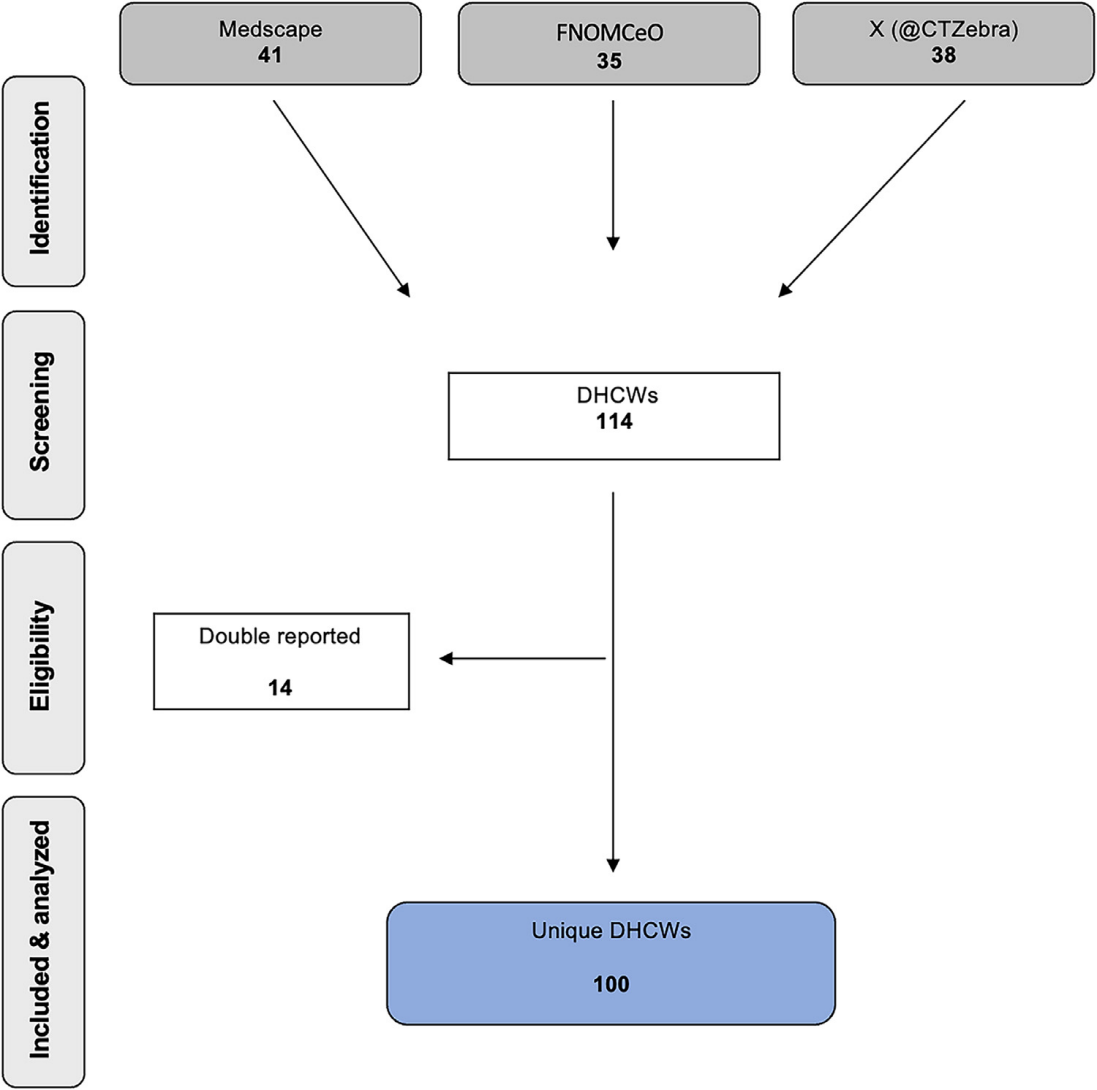
Flowchart of search results.

As reported in public-domain resources, 114 DHCWs were identified in the search conducted between March 2020 and May 2024. Among them, 14 DHCWs were found in more than one database. After removing these duplicates, 100 unique DHCWs were identified as having died due to the SARS-CoV-2 pandemic (see Figure 1 and Appendix A).

The DHCWs were based in 14 countries, with 40% of them located in the United States and 35% in Italy (Table 1).

**Table 1:** A global comparion of deaths of DHCWs who succumbed due to COVID-19.

**Country**	***N* (%)**
Canada	1 (1%)
Colombia	2 (2%)
Dominican Republic	1 (1%)
Ecuador	1 (1%)
Honduras	1 (1%)
Iran	1 (1%)
**Italy**	**35 (35%)**
Mexico	4 (4%)
Russia	1 (1%)
Turkey	5 (5%)
United Kingdom	2 (2%)
**United States**	**40 (40%)**
Venezuela	3 (3%)
Yemen	3 (3%)
**Total**	**100 (100%)**

The bold values are the highest of all values from the relevant table. The values at the bottom of the tables are also bold as total values.

The DHCWs could be categorized into eight distinct dental professions, with dentists comprising 79% (Table 2).

**Table 2:** DHCWs died due to COVID-19 presented by dental profession.

**Profession**	***N* (%)**
Dental assistant/nurse	5 (5%)
Dental hygienist	1 (1%)
Dental office manager	1 (1%)
Dental receptionist	4 (4%)
Dental technician	4 (4%)
**Dentist**	**79 (79%)**
Oral and maxillofacial/dental surgeon	4 (4%)
Orthodontist	2 (2%)
**Total**	**100 (100%)**

The bold values are the highest of all values from the relevant table. The values at the bottom of the tables are also bold as total values.

The ages of 43 deceased DHCWs were unavailable, whereas the ages of 57 subjects were reported (Appendix A). The mean age of the deceased DHCWs with a known age (*N* = 57) was 58 (sd = 12.70; range: 28-88). Among them, 49% were 60 to 70 years old (Table 3).

**Table 3:** Dental healthcare workers died due to COVID-19 presented by age category (*N* = 57).

**Age category**	***N* (%)**
20-30 y of age	2 (4%)
30-40 y of age	4 (7%)
40-50 y of age	9 (16%)
50-60 y of age	8 (14%)
**60-70 y of age**	**28 (49%)**
70-80 y of age	5 (9%)
80-90 y of age	1 (2%)
**Total**	**57 (100%)**

The bold values are the highest of all values from the relevant table. The values at the bottom of the tables are also bold as total values.

In addition, data from the X account @CTZebra^16^ indicated that the gender distribution was 30 (79%) men and eight (21%) women. In 62 cases Gender-API.com^17^ determined the gender based on the name. In total, 85 individuals (85%) were identified as male and 15 individuals (15%) as female in all three databases combined.

**Fig. 2:**
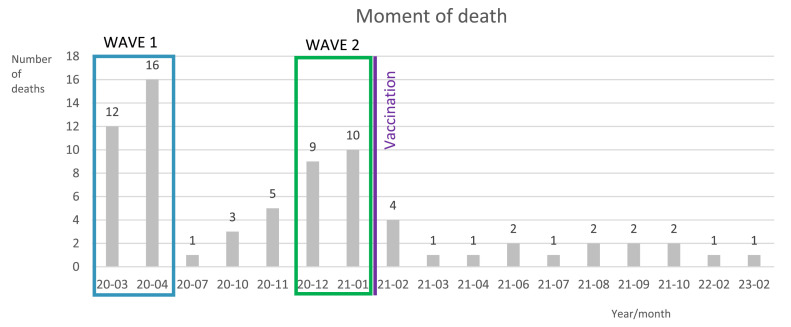
Illustration of dental healthcare workers died due to COVID-19, presented by moment of death (*N* = 73).

The date of death was provided in two databases, the X account @CTZebra^16^ and Medscape.^14^ Almost 50% of DHCWs died during the initial wave of the pandemic and during the subsequent second wave before the vaccination became available (Figure 2; for details, see Appendix B). No further deaths were identified after February 2023 on these databases.

## Discussion

This database document study evaluates the count of deceased DHCWs attributable to COVID-19 during the pandemic. Globally, 100 distinct DHCWs were registered in the public domain as deceased, maintaining the validity of their identities as healthcare professionals.

The cumulative number of deaths attributed to SARS-CoV-2 in Italy as of 10 March 2023, was 188,322. Among these fatalities, 34 Italian DHCWs lost their lives, constituting 0.018% of the deaths in Italy.^3^ In the United States, the total number of deaths due to COVID-19 as of 10 March 2023, was 1123,836. Among these, 40 American DHCWs died, representing 0.0036% of the deaths in the United States.^3^

According to WHO, Italy had 51,954 dentists in 2019,^19^ thus, approximately 0.065% of the dentists in Italy died during the pandemic. The United States reported 201,387 dentists and 546,830 dental assistants and practitioners in the same period.^19^ Among them, 0.0053% died due to COVID-19. In 2018, the global dentist population was estimated at 1615,539.^20^ In this population, the reported number of DHCWs (*N* = 100) who died from COVID-19, as reported in the public domain, was approximately 0.0061% of this population.

### COVID-19 in relation to gender

The European Centre for Disease Prevention and Control reported a male-to-female ratio of 2.1 for COVID-19 deaths across the European Union. Notably, the highest male-to-female ratio, reaching 3.9, among deaths was observed within the 50- to 65-year-old age group.^21^ A study conducted in England revealed a higher infection risk odds ratio of 1.55, interpreted as a small effect of COVID-19 among men compared to women.^22^^,^^23^ Consistent with these findings, the gender distribution of deceased individuals (where gender was known) in the current study was 79% male and 21% female. In cases where gender was estimated using the AI tool Gender-API.com,^17^ the distribution follows the pattern of 85% male and 15% female, suggesting this AI tool for gender was appropriate.

The skewed gender distribution could also be attributed to historical trends in the dental profession, where a higher proportion of men than women have been active over the past century.^24^ Although the percentage of women in the dental workforce has increased in the past 20 to 30 years, the age category with the highest number of deaths (60-70 years) primarily includes male workers. Thus, this demographic imbalance leads to a higher percentage of deceased men.

### DHCWs in the public domain

This study observed fatalities across 14 countries. The United States and Italy presented the highest number of deaths of DHCWs. However, this finding does not correspond to the countries with the highest overall death rates (per one million individuals) during the COVID-19 pandemic. Until 13 July 2022, Peru had the highest proportional death rate (6481.71 per million individuals).^25^ Moreover, Eastern Europe and South America nations experienced the highest proportional population loss. The United States (3099.62 per million) and Italy (2844.31 per million) are ranked 15th and 22nd, respectively, in the analysis.^25^ China, where the pandemic originated, reported a relatively low proportional population loss, with 10.38 deaths per million individuals. No deceased DHCWs from China were reported in the public domain.

### Vaccines

When examining the moment of death of the DHCWs, a clear wave is distinguishable (Figure 2). The number of deaths decreased after April 2020 and January 2021, likely due to the availability of vaccines.^26^^,^^27^ However, it is unknown whether the deceased were vaccinated. The reduction in deaths after the availability of vaccinations could be due to the end of the wave or vaccination itself.

### DHCWs in relation to age

Given that most of the deceased individuals with a known age were 60 years or older, it is reasonable to assume that some of them may not have been working as active DHCWs at the time of their deaths. However, this information was only confirmed for one individual (Appendix A: number 88, Stephen White). Across the European Union, the legal retirement age is around 65 years in most countries, ranging from 62 to 76 years.^28^ Nevertheless, recent data from the Organization for Economic Co-operation and Development indicates that many Europeans tend to leave the workforce earlier than the statutory retirement age.^28^ For instance, in Italy, which accounts for 35% of the total deceased individuals in this study, the mean retirement age was 56.8 years in 2009.^29^ However, the retirement age has an increasing trend.^30^ In the United States, the current mean retirement age is 67.^31^ Over a quarter (26.8%) of individuals over 65 remain active in the labour force. Based on these data, a portion of the deceased individuals 60 years or older were likely still actively engaged in the dental sector at the time of their passing.

### Infection prevention and control procedures

Numerous studies have explored oral healthcare safety and infection control practices during the COVID-19 pandemic. Dentists diligently adhere to strict infection control protocols, ensuring high safety for patients, dental team members, and themselves.^32^ In a specific European region with a notable number of COVID-19-related fatalities, dental professionals reported symptoms consistent with infection, especially in areas with a high prevalence of COVID-19. However, only a minimal proportion of participants received a confirmed diagnosis of COVID-19.^33^

Dentists in high-prevalence areas demonstrated lower adherence to precautionary measures than those in low-prevalence areas despite expressing a higher confidence level in avoiding infection. Although only one-third of dentists attended continuous educational courses on COVID-19, most felt they had adequate knowledge and used protective measures to prevent infection.^33^ Italian dental hygienists adapted their work practices to mitigate risks associated with the pandemic and displayed confidence in avoiding infection.^34^ Dentists’ experience managing diseases with comparable transmission mechanisms enabled them to adapt quickly, ensuring patient safety and reducing infection risk.^35^ Adherence to infection prevention and control procedures recommended by the Centers for Disease Control and Prevention significantly reduces infection risk in dental offices.^36^

Nonclinical activities and community transmission pose the most significant hazards for dentists exposed to COVID-19.^37^ Orthodontic providers have implemented effective mitigation strategies, resulting in low infection rates.^38^ Despite pandemic challenges, dentists and dental hygienists have maintained confidence in their professions.^39^ Although enhanced infection control measures were reported in most dental practices, the consistent use of personal protective equipment by dental hygienists remains an area for improvement.

Data collection can help estimate COVID-19 incidence rates among dental hygienists in the United States and identify the associated risk factors.^40^ Dental offices in New York reported no instances of infection among dentists, staff, or patients, even with a high proportion of high-risk comorbidities.^41^ In the Netherlands, oral healthcare providers adhered to COVID-19 modifications to national guidelines, resulting in infection rates similar to other healthcare workers.^10^ They also intensely focused on hygiene and infection control measures, resulting in a low incidence of COVID-19 infections.^42^

### Strengths

A series of multivariable-adjusted analyses conducted on cohorts of patients with COVID-19 indicated a significant association between higher disease severity and demographic factors, including older age and male gender.^43^ Other studies have also confirmed that increased age was associated with death in patients with COVID-19.^44^^,^^45^ The results of these studies align with the data observed in this study; thus, the results are generalizable.

### Limitations

In this study, the fatalities were observed across 14 countries. This is only 7% of the countries while the pandemic was worldwide. The DHCW deaths from the continents of Africa and Australia were not found in the datasets. According to Medscape,^14^ the registration of deceased healthcare workers relied on contributions from colleagues, friends, and family members of those who died. Therefore, the use of public-domain databases results in an underestimation of the data. The registration list, which encompasses over 1800 names from 64 countries, is acknowledged to be incomplete.

Medscape^14^ also acknowledged that, in some instances, the list includes the names of individuals who did not die from COVID-19 but whose deaths were associated with the stress and challenges imposed by the pandemic, contributing to an overestimation of the data. Furthermore, older individuals from the registration lists were possibly retired and may not have been infected with COVID-19 through contact with patients but through social contact. In addition, those on the list could have died due to general health complications unrelated to COVID-19. Moreover, those with pre-existing health conditions were more susceptible to mortality due to a COVID-19 infection.^46^

Additionally, Medscape^14^ stated that they were unable to include names without confirmation of COVID-19-related death. This limitation also applies to the other two databases in this study. Two databases^15^^,^^16^ were used, where the author or publishing authority was located in Italy and the United States, which could have distorted the result, because these databases may have focused on residents. In addition, family, friends, and colleagues were aware that the death of the DHCW could be reported to these databases.

Although it is impossible to provide a comprehensive and adequate representation of the entire population of DHCWs worldwide who died solely due to COVID-19, this study is a realistic approach for an estimation.

### Implications for clinical practice and future research

A recommendation is for national societies of DHCWs to maintain comprehensive records of the status of their professions during a pandemic. For instance, the Royal Dutch Dental Association for Dentists could compile and archive relevant data in the Netherlands. Thus, international organizations, such as the World Dental Federation and WHO, could accurately estimate the absolute influence of the pandemic on the dental profession. This proactive approach to data collection and documentation could facilitate a better understanding of the challenges facing DHCWs during future pandemics and aid in the developing targeted strategies to address these challenges effectively.

## Conclusion

During the global pandemic, DHCWs operated in the framework of general practice. As part of their infection prevention measures, they adhered to standard guidelines and incorporated new adjunctive measures designed explicitly for the pandemic, such as the COVID-19 guideline for oral healthcare. International public registers documented that 100 DHCWs died, due to the pandemic. Of these cases, the majority were men between 60 and 70 years. The United States and Italy reported the highest number of deceased DHCWs. Considering the limited number of fatalities among DHCWs reported in the public domain, it measures in dental practices provide adequate protection, as the mortality rate was less than 0.006% of their population.

## Ethics statement

The protocol was registered at the ACTA by 2022-52198.

## Data availability statement

The data that support the findings of this study are available from the corresponding author upon reasonable request.

## Disclaimer

This manuscript includes references to individuals whose names have been obtained from publicly available databases. The privacy and reputation of these individuals are duly acknowledged and protected.

## Author contributions

Marlotte C. van Capelleveen and Nadine Jaquet: Contributed to design, data collection, analysis, interpretation of the data and critically drafted the manuscript. Thérèse A. Elkerbout, Eveline van der Sluijs, and Dagmar Else Slot: Contributed to conception and design, analysis and interpretation, and critically revised the manuscript. All authors gave final approval and consented to be accountable for all aspects of work, ensuring its integrity and accuracy.

## Conflict of interest

The authors declare that they have no conflicts of interest.

## Appendix Supplementary materials

Image, application 1

## Footnotes

Supplementary material associated with this article can be found in the online version at doi:10.1016/j.identj.2024.10.012.
