# Supplementary material for: Mortality Among Dental Healthcare Workers During the Coronavirus Disease 2019 Pandemic: A Public Domain Database Study
Source: Int Dent J. 2024 Dec 18;75(2):692–9. doi: 10.1016/j.identj.2024.10.012 (PMC11976538; doi:10.1016/j.identj.2024.10.012)
Supplement: Supplementary file 1 [file mmc1.docx]

**APPENDICES**
**Appendix A.** Total overview of dental healthcare workers died due to COVID-19 presented with full name, profession, country, age, gender, date of death and data source.

| **#** | **Name** | **Surname** | **Profession** | **Country** | **Age** | **Gender^a^** | **Moment of death** | **Data source** |
| --- | --- | --- | --- | --- | --- | --- | --- | --- |
| 1. | Aaron | Perez | Dentist | US | unknown | M | 31-12-2020 | X @CTZebra |
| 2. | Abdulghani | Taki Makki | Dentist | Italy | 78 | M^b^ | 24-3-2020 | Medscape, FNOMCeO |
| 3. | Adel | Al-Sarari | Dentist | Yemen | unknown | M^b^ | unknown | Medscape |
| 4. | Aileen | Sartaguda | Dental surgeon | UK | unknown | F^b^ | unknown | Medscape |
| 5. | Alan | Kratenstein | Dentist | US | 63 | M | 13-4-2020 | X @CTZebra |
| 6. | Angela "Angie" | Dobbs | Dental receptionist | US | 41 | F | 17-1-2021 | X @CTZebra |
| 7. | Antonio | de Pisapia | Dentist | Italy | unknown | M^b^ | 6-4-2020 | Medscape, FNOMCeO |
| 8. | Armando | Crisafulli | Dentist | Italy | unknown | M^b^ | 02-02-2021 | FNOMCeO |
| 9. | Ashok | Patel | Dentist | US | 63 | M | 25-12-2020 | X @CTZebra |
| 10. | Bill | Marinakas | Dentist | US | 53 | M | 9-9-2021 | X @CTZebra |
| 11. | Brandon | Taylor | Dental technician | US | 28 | M | 18-7-2020 | X @CTZebra |
| 12. | Bulent | Azman | Dentist | Turkey | 61 | M^b^ | unknown | Medscape |
| 13. | Cheryl | Patterson | Dentist | US | 63 | F | 28-2-2021 | X @CTZebra |
| 14. | Chester | Banaag | Dentist | US | 57 | M | 1-1-2021 | X @CTZebra |
| 15. | Chris | Firlit | Oral and maxillofacial surgeon | US | 37 | M^b^ | unknown | Medscape |
| 16. | Chris | Jernigan | Orthodontist | US | 67 | M | 7-8-2021 | X @CTZebra |
| 17. | Curtis | Orr | Dental technician | US | 55 | M | 9-4-2020 | X @CTZebra |
| 18. | Daryn | Moody | Dentist | US | 62 | M | 29-8-2021 | X @CTZebra |
| 19. | Denis | Vincent | Dentist | Canada | 62 | M^b^ | unknown | Medscape |
| 20. | Domenico | Bardelli | Dentist | Italy | 75 | M^b^ | 20-3-2020 | Medscape, FNOMCeO |
| 21. | Domenico | Fatica | Dentist | Italy | unknown | M^b^ | 13-4-2020 | Medscape, FNOMCeO |
| 22. | Eli | Landau | Dentist | US | 60 | M | 5-4-2020 | X @CTZebra |
| 23. | Emanuele | Salvatore | Dentist | Italy | unknown | M^b^ | 01-06-2021 | FNOMCeO |
| 24. | Enrico | Boggio | Dentist | Italy | unknown | M^b^ | 7-4-2020 | Medscape, FNOMCeO |
| 25. | Enrique Macedo | Orta | Dentist | Mexico | unknown | M^b^ | unkown | Medscape |
| 26. | Esmail | Yazdi | Oral and maxillofacial surgeon | Iran | unknown | M^b^ | unknown | Medscape |
| 27. | Ferdinando | de Crescenzo | Dentist | Italy | unknown | M^b^ | 22-12-2020 | FNOMCeO |
| 28. | Fouad Ahmed | Moqbel | Dentist | Yemen | unknown | M^b^ | unknown | Medscape |
| 29. | Gabriele | Lombardi | Dentist | Italy | 68 | M^b^ | 18-3-2020 | Medscape, FNOMCeO |
| 30. | Gary "Mark" | Davis | Dentist | US | 61 | M | 11-1-2021 | X @CTZebra |
| 31. | Gerardo Palacios | Jiménez | Dentist | Mexico | unknown | M^b^ | unknown | Medscape |
| 32. | Gianroberto | Monti | Dentist | Italy | unknown | M^b^ | 21-3-2020 | Medscape, FNOMCeO |
| 33. | Giovanni | Stagnati | Dentist | Italy | 71 | M^b^ | 22-3-2020 | Medscape, FNOMCeO |
| 34. | Giovanni | Cerchiello | Dentist | Italy | unknown | M^b^ | 25-3-2020 | FNOMCeO |
| 35. | Giunio | Matarazzo | Dentist | Italy | unknown | M^b^ | 7-4-2020 | Medscape, FNOMCeO |
| 36. | Giuseppe | de Vita | Dentist | Italy | unknown | M^b^ | 15-1-2021 | FNOMCeO |
| 37. | Giuseppe | Lauriola | Dentist | Italy | unknown | M^b^ | 20-12-2020 | FNOMCeO |
| 38. | Guilo | de Carli | Dentist | Italy | unknown | M^b^ | 3-2-2021 | FNOMCeO |
| 39. | Halil | Sarikaya | Dental technician | Turkey | unknown | M^b^ | unknown | Medscape |
| 40. | Hamza | Zaid | Dentist | Italy | unknown | M^b^ | 12-02-2023 | FNOMCeO |
| 41. | Héctor | Morfe | Dentist | Venezuela | 40 | M^b^ | unknown | Medscape |
| 42. | Henry | Tong | Dentist | US | 61 | M | 23-2-2021 | X @CTZebra |
| 43. | Ignazio Alberto Gómez | Alzate | Dentist | Colombia | 58 | M^b^ | unknown | Medscape |
| 44. | Imelda | Torres | Dentist | Mexico | ? | F^b^ | unknown | Medscape |
| 45. | Ivano | Garzena | Dentist | Italy | 48 | M^b^ | 23-3-2020 | Medscape, FNOMCeO |
| 46. | James "Jimmy" | Jordan | Dentist | US | 61 | M | 9-6-2021 | X @CTZebra |
| 47. | Jaqueline | Wheeler | Dental receptionist | US | 48 | M | 11-6-2021 | X @CTZebra |
| 48. | Jeffory | Eaton | Dentist | US | 69 | M | 15-3-2021 | X @CTZebra |
| 49. | John | Jordan | Dentist | US | 88 | M | 21-1-2021 | X @CTZebra |
| 50. | Karen | Kelly | Dental assistant | US | 62 | F | 4-4-2020 | X @CTZebra |
| 51. | Kassem | el Malak | Dentist | Italy | unknown | M^b^ | 23-11-2020 | FNOMCeO |
| 52. | Kenneth | Jordan | Dental technician | US | 66 | M | 11-11-2020 | X @CTZebra |
| 53. | Leonardo | Ditta | Dentist | Italy | unknown | M^b^ | 2-9-2021 | FNOMCeO |
| 54. | Linnette | Cruz | Dental Nurse | UK | 51 | F^b^ | unknown | Medscape |
| 55. | Lino Gordon | Rodríguez | Dentist | Colombia | 63 | M^b^ | unknown | Medscape |
| 56. | Luca | Parolari | Dentist | Italy | unknown | M^b^ | 01-07-2021 | FNOMCeO |
| 57. | Mabel | Zùñiga de Logacho | Dentist | Ecuador | 48 | F^b^ | unknown | Medscape |
| 58. | Marco | Lera | Dentist | Italy | 68 | M^b^ | 20-3-2020 | Medscape, FNOMCeO |
| 59. | María Mercedes Molina | Roa | Dentist | Venezuela | 37 | F^b^ | unknown | Medscape |
| 60. | Mario | Calonghi | Dentist | Italy | 55 | M^b^ | 22-3-2020 | Medscape, FNOMCeO |
| 61. | Mario | Ronchi | Dentist | Italy | unknown | M^b^ | 20-3-2020 | Medscape, FNOMCeO |
| 62. | Maurizio | Abatini | Dentist | Italy | unknown | M^b^ | 19-10-2020 | FNOMCeO |
| 63. | Maurizio | Gasparini | Dentist | Italy | unknown | M^b^ | 01-02-2021 | FNOMCeO |
| 64. | Mauro | Cotillo | Dentist | Italy | unknown | M^b^ | 19-11-2020 | FNOMCeO |
| 65. | Messer | Ahmed | Dentist | US | 64 | M | 18-1-2021 | X @CTZebra |
| 66. | Miriam Arlet | Diaz | Dental receptionist | US | 29 | F | 26-2-2022 | X @CTZebra |
| 67. | Mohammad Alì | Zaraket | Dentist | Italy | unknown | M^b^ | 2-12-2020 | FNOMCeO |
| 68. | Neaunce "Nika" | Vilcina | Dental office manager | US | unknown | F | 10-02-2021 | X @CTZebra |
| 69. | Nicola | Cocucci | Dentist | Italy | unknown | M^b^ | 8-4-2020 | FNOMCeO |
| 70. | Óscar | Villa Garza | Dentist | Mexico | unknown | M^b^ | unknown | Medscape |
| 71. | Paolo | Duso | Dentist | Italy | unknown | M^b^ | 12-09-2020 | FNOMCeO |
| 72. | Patrick Jr. | Kildea | Dentist | US | 77 | M | 2-4-2021 | X @CTZebra |
| 73. | Rafael Reyes | Lantigua | Dentist | Dominican Republic | unknown | M^b^ | unknown | Medscape |
| 74. | Randall | Pierce | Dentist | US | 69 | M | 25-12-2020 | X @CTZebra |
| 75. | Riccardo | Scarduelli | Dentist | Italy | unknown | M^b^ | 22-7-2021 | FNOMCeO |
| 76. | Richard | Compton | Dentist | US | 66 | M | 3-12-2020 | X @CTZebra |
| 77. | Robert "Rob" | Burnett | Dentist | US | 63 | M | 2-2-2021 | X @CTZebra |
| 78. | Roberto | Governi | Dentist | Italy | unknown | M^b^ | 11-10-2020 | FNOMCeO |
| 79. | Roberto | Zambonin | Dentist | Italy | unknown | M^b^ | 10-12-2020 | FNOMCeO |
| 80. | Rodney | Mack | Dental receptionist – Public safety officer at NYU College of Dentistry | US | 54 | M | 5-4-2020 | X @CTZebra |
| 81. | Ruben | Moronta | Dentist | US | 54 | M | 6-4-2020 | X @CTZebra |
| 82. | Sam | Worthington IV | Dentist | US | 48 | M | 13-11-2020 | X @CTZebra |
| 83. | Scott | Blanks | Dental assistant | US | 34 | M | 27-3-2020 | X @CTZebra |
| 84. | Serkan | Munis | Dentist | Turkey | 43 | M^b^ | unknown | Medscape |
| 85. | Seyfi | Gur | Dentist | Turkey | unknown | M^b^ | unknown | Medscape |
| 86. | Shawna | Berreman | Dental assistant (XRay technician) | US | 49 | F | 17-3-2020 | X @CTZebra |
| 87. | Silverio "Sil" | Mazzella | Dentist | US | 60 | M | 3-4-2020 | X @CTZebra |
| 88. | Stephan | White | Pediatric Dentist | US | 74 | M | 26-4-2020 | X @CTZebra |
| 89. | Steven | Huber | Orthodontist | US | 62 | M | 3-4-2020 | X @CTZebra, Medscape |
| 90. | Tatyana | Safonova | Dentist | Russia | 67 | F^b^ | unknown | Medscape |
| 91. | Theodore | Corcoran | Dentist / Oral and maxillofacial surgeon | US | 61 | M^b^ | unknown | Medscape |
| 92. | Tina | Boghossian | Dental hygienist | US | 34 | F | 2-10-2021 | X @CTZebra |
| 93. | Tomás | Canales | Dentist | Honduras | unknown | M^b^ | unknown | Medscape |
| 94. | Tommaso | di Loreto | Dentist | Italy | unknown | M^b^ | 13-4-2020 | FNOMCeO |
| 95. | Turk Mustafa | Oral | Dentist | Turkey | 68 | M^b^ | unknown | Medscape |
| 96. | Vittorio | Collesano | Dentist | Italy | unknown | M^b^ | 31-10-2020 | FNOMCeO |
| 97. | Wanda "Kay" | Worley | Dental assistant | US | 67 | F | 22-1-2021 | X @CTZebra |
| 98. | William "Bill" | Cohen | Dentist | US | 69 | M | 14-4-2020 | X @CTZebra |
| 99. | Yolimar | Márquez de Salgado | Dentist | Venezuela | 48 | F^b^ | unknown | Medscape |
| 100. | Yousry | Mejalli | Dentist | Yemen | unknown | M^b^ | unknown | Medscape |

^a^ Gender: Male (M), Female (F)

^b^ Based on the outcome of Gender-API

Disclaimer:

The GDPR (General Data Protection Regulation) is respected; however, the names of individuals included in this thesis are obtained from public resources.

**Appendix B.** Dental healthcare workers died due to COVID-19 presented per month

| **Moment of death (year-month)** | **N (%)** |
| --- | --- |
| **March 2020 (20-03)** | **12 (16%)** |
| **April 2020 (20-04)** | **16 (22%)** |
| July 2020 (20-07) | 1 (1%) |
| October 2020 (20-10) | 3 (4%) |
| November 2020 (20-11) | 5 (7%) |
| **December 2020 (20-12)** | **9 (12%)** |
| **January 2021 (21-01)** | **10 (14%)** |
| February 2021 (21-02) | 4 (5%) |
| March 2021 (21-03) | 1 (1%) |
| April 2021 (21-04) | 1 (1%) |
| June 2021 (21-06) | 2 (3%) |
| July 2021 (21-07) | 1 (1%) |
| August 2021 (21-08) | 2 (3%) |
| September 2021 (21-09) | 2 (3%) |
| October 2021 (21-10) | 2 (3%) |
| February 2022 (22-02) | 1 (1%) |
| February 2023 (23-02) | 1 (1%) |
| **Total** | **73 (100%)** |
